# Towards more personalized digital health interventions: a clustering method of action and coping plans to promote physical activity

**DOI:** 10.1186/s12889-022-14455-4

**Published:** 2022-12-12

**Authors:** Helene Schroé, Stéphanie Carlier, Delfien Van Dyck, Femke De Backere, Geert Crombez

**Affiliations:** 1 Department of Experimental-Clinical and Health Psychology, Faculty of Psychology and Educational Sciences, Ghent University, Ghent, Belgium; 2 Department of Movement and Sports Sciences, Faculty of Medicine and Health, Research Group Physical Activity and Health, Ghent University, Watersportlaan 2, 9000 Ghent, Belgium; 3 IDLab, Department of Information Technology, Ghent University—imec, Technologiepark-Zwijnaarde 126, 9052 Ghent, Belgium

**Keywords:** Digital health, E-health, M-health, Behaviour change, Physical activity, Personalization, Knowledge-base, Decision support systems

## Abstract

**Background:**

Despite effectiveness of action and coping planning in digital health interventions to promote physical activity (PA), attrition rates remain high. Indeed, support to make plans is often abstract and similar for each individual. Nevertheless, people are different, and context varies. Tailored support at the content level, involving suggestions of specific plans that are personalized to the individual, may reduce attrition and improve outcomes in digital health interventions. The aim of this study was to investigate whether user information relates toward specific action and coping plans using a clustering method. In doing so, we demonstrate how knowledge can be acquired in order to develop a knowledge-base, which might provide personalized suggestions in a later phase.

**Methods:**

To establish proof-of-concept for this approach, data of 65 healthy adults, including 222 action plans and 204 coping plans, were used and were collected as part of the digital health intervention MyPlan 2.0 to promote PA. As a first step, clusters of action plans, clusters of coping plans and clusters of combinations of action plans and barriers of coping plans were identified using hierarchical clustering. As a second step, relations with user information (i.e. gender, motivational stage, ...) were examined using anova’s and chi^2^–tests.

**Results:**

First, three clusters of action plans, eight clusters of coping plans and eight clusters of the combination of action and coping plans were identified. Second, relating these clusters to user information was possible for action plans: 1) Users with a higher BMI related more to outdoor leisure activities (F = 13.40, *P* < .001), 2) Women, users that didn’t perform PA regularly yet, or users with a job related more to household activities (X^2^ = 16.92, *P* < .001; X^2^ = 20.34, *P* < .001; X^2^ = 10.79, *P* = .004; respectively), 3) Younger users related more to active transport and different sports activities (F = 14.40, *P* < .001). However, relating clusters to user information proved difficult for the coping plans and combination of action and coping plans.

**Conclusions:**

The approach used in this study might be a feasible approach to acquire input for a knowledge-base, however more data (i.e. contextual and dynamic user information) from possible end users should be acquired in future research. This might result in a first type of context-aware personalized suggestions on the content level.

**Trial registration:**

The digital health intervention MyPlan 2.0 was preregistered as a clinical trial (ID:NCT03274271). Release date: 6-September-2017.

**Supplementary Information:**

The online version contains supplementary material available at 10.1186/s12889-022-14455-4.

## Background

Promoting a healthy lifestyle is key in reducing the burden of non-communicable diseases such as type 2 diabetes, cancer, osteoarthritis, depression and cardiovascular diseases [1, 2]. Digital health interventions, an umbrella term for the usage of digital technology to support health [3], can be employed to promote a healthy lifestyle and has gained popularity because of its time- and cost-effectiveness [4–6].

Digital health interventions have been found to be more effective when informed by a behaviour change theory in comparison with a-theoretical interventions [7]. Several theories have been developed (e.g. social cognitive theory, the health belief model, self-determination theory), but one of the most comprehensive models is the Health Action Process Approach (HAPA) model [8]. The HAPA-model is a two-phase model that guides individuals to change their behaviour, beginning with the development of an intention (motivational phase), followed by bridging the gap between intention and the actual behaviour (volitional phase) [8, 9]. Behaviour change techniques (BCTs) such as *action planning* (i.e. where participants select their own goals and decide what they want to do & how, where and when they want to do it) and *coping planning* (i.e. exploring solutions for possible barriers) are key components within the HAPA-model to bridge the intention-behaviour gap [10–13].

Despite the effectiveness of action and coping plans in digital health interventions to change behaviour [14–16], attrition rates remain high [14, 17, 18]. This reduces the impact of these interventions. An important reason for these high attrition rates might be that support offered by digital interventions to make such action and coping plans usually is abstract, generic and the same for each individual (a so-called “one size fits all intervention”) [14]. Nevertheless, people are different, and the context in which they behave varies between individuals [19]. There is a need to provide support in digital health interventions in a more personalized and contextualized way.

As yet, interventions provide already tailoring at the construct level or BCT level (e.g. participants who have an intention to change for example receive other BCTs than participants who do not have an intention to change) [20]. However, practical support on the content level (i.e. concrete operationalizations of BCTs such as action and coping planning), is not provided in a personalized and contextualized way. In previous studies [14, 16, 17], participants were considered as their own expert in terms of making plans: they specify themselves the content of their plans and take their own personal and context-factors into account. Nevertheless, this approach resulted in a low quality of plans, and participants experienced difficulties in formulating them [21, 22]. As a result, support at the content level is needed: this support should include suggestions of specific plans that are personalized to the individual (e.g. If someone is retired, he should not get the advice to walk to work) and contextualized to the individual (e.g. If someone is working from home, she should not get the advice to go for a lunch walk with a colleague).

A promising approach is to use intelligent algorithms and decision support systems [23]. The term “decision support system” (=DSs) is a broad concept covering all aspects of support during decision making, and provides automated recommendations where required and when available [23]. As such, a DSs could improve tailoring in digital health interventions by suggesting a relevant plan to do physical activity (PA) that is personalized and contextualized to the individual. Notwithstanding the potential of a DSs, a knowledge-base should first be developed in order to deliver such suggestions of plans [23, 24]. A knowledge-base is defined as a collection of facts, assertions, relationships, rules about a specific domain represented in a computer readable format [24]. The process of acquiring knowledge for the knowledge-base is defined as knowledge acquisition and may come from multiple sources (experts, books, research findings, etc) [24]. For our purpose, the knowledge-base should at least contain relationships between personal and contextual user information (i.e. information that relates to the individual itself such as demographic information, motivational stage, emotions; information that relates to the context of the individual such as physical and social environment, the weather, respectively) and PA plan characteristics (e.g. PA type, place of the activity, time of the activity, barriers to do the activity). Once a knowledge-base is developed, it may become possible to deliver context-aware personalized suggestions to a user, as the DSs can exploit the knowledge-base to find appropriate suggestions based on specific user information of that user (e.g. if a user is a female younger adult living with her partner in a rural environment and the knowledge-base contains relationships between these characteristics and certain outdoor physical activities, these activities can then be delivered as a suggestion to the user).

The main objective of this study is to empirically investigate whether user information relates toward specific action and coping plans (e.g. motivated users may rather plan vigorous physical activities compared to less motivated users; adults who work may rather experience barriers such as not having time for PA compared to retired adults). More specifically, this paper will rather address personal user information than contextual user information. As such, this paper provides a proof-of-concept on how knowledge can be acquired in order to develop such a knowledge-base. This includes a clustering method with a two-steps approach. The first step is to explore whether patterns in action and coping plans can be identified using clustering algorithms on available data. The second step is to examine whether these clusters of action and coping plans can be linked to specific user information.

## Methods

### Data source

Data was used from the ‘MyPlan 2.0’ factorial randomized controlled trial, which was conducted between February 2018 and December 2018, and was approved by the Ghent University Hospital Ethics Committee (ID number: NCT03274271). The protocol paper of ‘MyPlan 2.0’ can be found elsewhere [25].

### MyPlan 2.0

#### Intervention

‘MyPlan 2.0’ was a digital health intervention that consisted of a website and an optional mobile application to promote PA in healthy adults from the general population. ‘MyPlan 2.0’ was based on the HAPA-model and consisted of a number of BCTs to guide participants in changing their behaviour. The BCTs used in ‘MyPlan 2.0’ were goal setting, providing information on consequences of behaviour, providing feedback on performance, social support, action planning, coping planning, self-monitoring and reviewing behaviour goals. The intervention consisted of 5 website sessions, with 1 week between each session. In each website session, participants were prompted to create their own action and coping plans in order to reach their PA goal. The app was synchronized with the website and was offered to participants as an extension to support users in their plans on a daily basis. The usage of the app was optional. It consisted of different modules through which participants could freely navigate (i.e. a quiz module regarding benefits of more PA, an action plan module, a coping plan module, a self-monitoring module, a gamification module). For example, in the action and coping planning modules, participants could review the action and coping plans that they created on the website, and could change these plans throughout the week. The app also reminded participants of their plan by sending a notification on the scheduled moment. More information about the website and app can be found in the protocol paper of MyPlan [25].

#### Procedure and participants

Before starting with the intervention, participants had to complete a pre-test questionnaire, assessing 1) demographic variables, 2) psychosocial determinants of behaviour change including motivational stage, and 3) their current PA level. When the pre-test measurements were completed, participants were randomly allocated to different versions of the intervention as part of the design of the ‘MyPlan 2.0’ factorial randomized controlled trial. They were allocated to eight different groups to evaluate the efficacy of three BCTs (i.e. action planning, coping planning and self-monitoring) and their combinations. As such, each group received a different version of the digital health intervention, including different BCTs. Therefore, only data of participants allocated to the groups who received both the BCTs action and coping planning, were included in this study. As such, data of 65 participants were used, including 222 action plans and 204 coping plans. Inclusion criteria were [25]: (1) having a minimum age of 18, (2) speaking Dutch, (3) having internet access and being the owner of a smartphone (iOS or Android). Participants were excluded if a risk for adverse effects during physical activity was detected. For that purpose, participants completed the ‘Physical Activity Readiness Questionnaire’ (PAR-Q) [26], and were excluded when they answered “yes” on one of the seven questions.

### Measures

The data in this paper includes 1) the created action and coping plans and 2) specific user information.

#### Action and coping plans

**Fig. 1:**
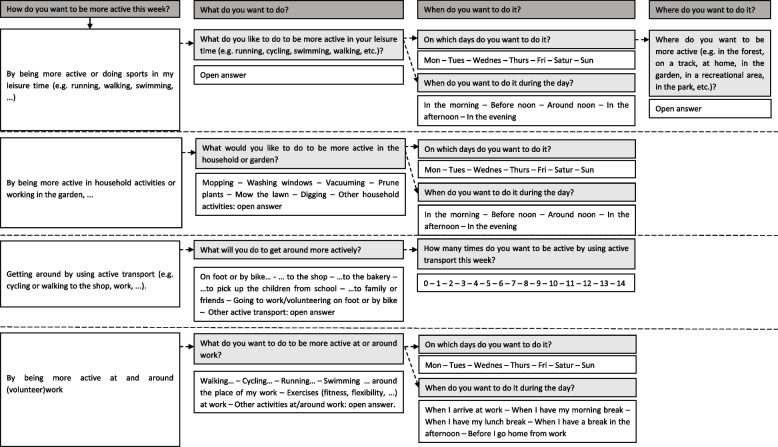
Questions and answer options for the action plans

**Fig. 2:**
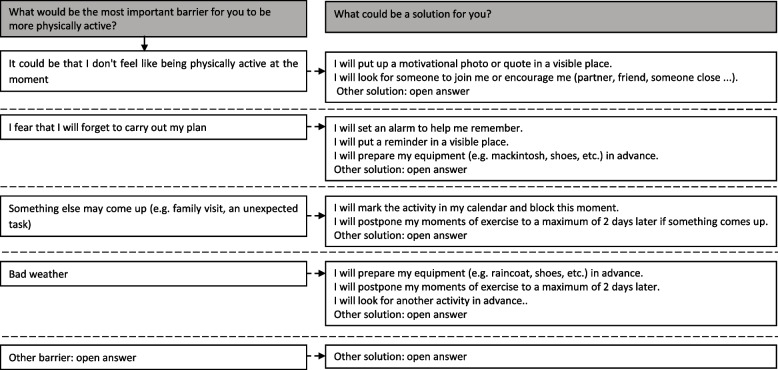
Questions and answer options for the coping plans

Once a week in each website session, participants were prompted to create their own action and coping plans. For the BCT action planning, participants could create a plan by specifying how they wanted to be more physically active, what they wanted to do, where and when they wanted to do it. Figure 1 shows an overview of the questions asked with possible answer options in order to guide participants in the process of action planning. For the BCT coping planning, participants had to identify difficult situations or barriers they anticipated to experience while being more physically active in the upcoming week. Participants were then prompted to think about a relevant solution for their barrier. In order to guide participants in the process of coping planning, a list of possible barriers and their solutions were provided, which is shown in Fig. 2.

#### User information

Specific user information in this paper addressed personal user information (i.e. information related to the individual itself) including the demographic information and motivational stage of participants. The following demographic variables were used for analyses: age (continuous), height and weight to calculate BMI (continuous), gender (categorized as male and female), educational level (categorized as not having versus having a college/university degree), occupation (categorized as having a job versus not having a job) and marital status (categorized as having a partner versus not having a partner). Motivational stage was measured using items of the HAPA-model [8, 13] and was categorized as pre-intenders (i.e. participants who had no intention to change their behaviour), intenders (i.e. participants who already developed an intention to change their behaviour but did not perform PA regularly yet) and actors (i.e. participants who already perform PA regularly).

### Data analysis

A clustering method with a two steps-approach was used. The first step was to explore whether patterns in action and coping plans could be identified using clustering algorithms. The second step was to examine whether these clusters of action and coping plans could be linked to specific user information.

#### Coding action and coping plans

Before conducting the clustering analyses (i.e. the first step), the action and coping plans were coded to be computer readable. As a pre-processing step, the different action plans for the same user were separated into individual samples. Furthermore, entries were separated such that one action plan concerns only one of the four PA domains, i.e. sports or leisure, household activities, active transport or (volunteer)work. Next, action and coping plans were coded into different variables, based on the answering options of Figs. 1 & 2 and the most frequent open answers that were given by participants. Table 1 shows an overview of these most frequent open answers.

**Table 1:** Overview of the most frequent open answers within MyPlan

Answering options of Figs. 1 & 2 that required an open answer	Open answers that were used as variables for clustering (n)		*Answers of the variable “other” (n)	
*Action plan – sports or leisure time activities*	Walking (47), Biking (49), Running (62), Swimming (21), Work-out (4),	Yoga (13), Fitness (25), Dancing (1), Tennis (13), Other sports* (9)	Longboarding (2)^a^, Rope skipping (5)^a^, Skeelering (1)^a^, Canoeing (1) ^a^	
*Action plan – other household activities*	Weeding (1)	Other gardening* (6)	Cleaning terrace (4)^a^, Rough gardening (2)^b^	
*Action plan – other active transport*	Other active transport* (4)		Going to hobby (4x)^a^	
*Action plan – other moving at work*	Taking the stairs at work (1),	Other activities at/around work* (1)	Cleaning the office (1)^a^,	
*Coping plan – other barriers*	No time (62), Tired (12), Don’t feel like doing it alone (6), Sick (6),	Pain (4), No barriers (8), Other barriers* (8)	Stress (2)^a^, Getting sweaty (1)^a^, Having kids home (1)^a^, Too much people around (1)^a^,	Bad tempered (1)^b^ Obsessive-compulsive disorder (1)^a^ Searching for excuses (1) ^b^
*Coping plan – other solutions*	Planning (34), Manage pain (by for example medication) (1), Give yourself a reward (6),	No solution (9), Other solutions* (13)	Making time (3)^b^, Just do it (1)^b^, Shortening the activity (2)^a^, Taking a shower (1)^a^, Realizing being active does not take up much time (1)^a^,	Don’t forget (1)^b^, Prepare the kids for doing the activity (1)^a^, Don’t overthink (1)^b^, Search motivation (1)^b^, Drinking water (1)^a^,

*n* number of times this option was chosen by participants to create their action and coping plan *These answers of participants were used as the variable “other” for clustering because they were not frequently chosen^a^ or the answer was considered less qualitative^b^

All variables of the action and coping plan that were coded can be found in Additional file 1. A “0” was coded if the variable was not applicable, a “1” was coded if the variable was applicable. All these variables of the action and coping plan that were coded can also be found in the second column of Figs. 6 and 7 respectively.

#### Clustering analysis

To explore whether patterns in action and coping plans could be identified, clustering algorithms were used. More specifically, hierarchical clustering was used to identify clusters of action and coping plans. Clustering analysis was conducted using custom Python software, based on the skicit-learn package [27], a machine learning library. Only the information regarding the action and coping plans of the users were included in this analysis.

Hierarchical clustering analyses were conducted to identify 1) clusters of action plans (*including all the variables of the action plans*), 2) clusters of coping plans (*including all the variables of the coping plans*) and 3) clusters of the combination of action and coping plans. For the clustering of the combination of action and coping plans, a subset of variables of these plans is used. More specifically, for the action plans, the domain of PA relevant to the action plan and the variables that relate to the users’ possible context when performing the listed activity, e.g., day of the week, time of day, location, and for the coping plans the subset of barriers is used. This, with the objective to investigate the link between action and coping plans, and more specifically to investigate if the user’s current context is linked to the barriers they might experience for that specific planned activity. The hierarchical clustering analyses have been performed using the Hamming distance as a dissimilarity measure and complete-linkage as the linkage criterion to calculate inter-cluster distances and define the optimal number of clusters [28]. In complete-linkage clustering, the distance between two clusters is considered as the distance between the two vectors furthest away from each other in each cluster.

To evaluate the density and separation between the resulting number of clusters, Silhouette Analysis has been performed for a range of possible cluster numbers [29]. This Silhouette Analysis gives information on the optimal number of clusters and how well a sample has been clustered. Samples with a Silhouette Coefficient (SC) close to 1 are very well clustered, a SC close to 0 indicates the sample lies in between two clusters and a sample with a negative SC has probably been placed in the wrong cluster. Overall, for the optimal number of clusters, clusters do not have a SC below the average SC and the resulting clusters are preferably similar in size. Nonetheless, expert input is needed to validate that the formed clusters are logical.

**Fig. 3:**
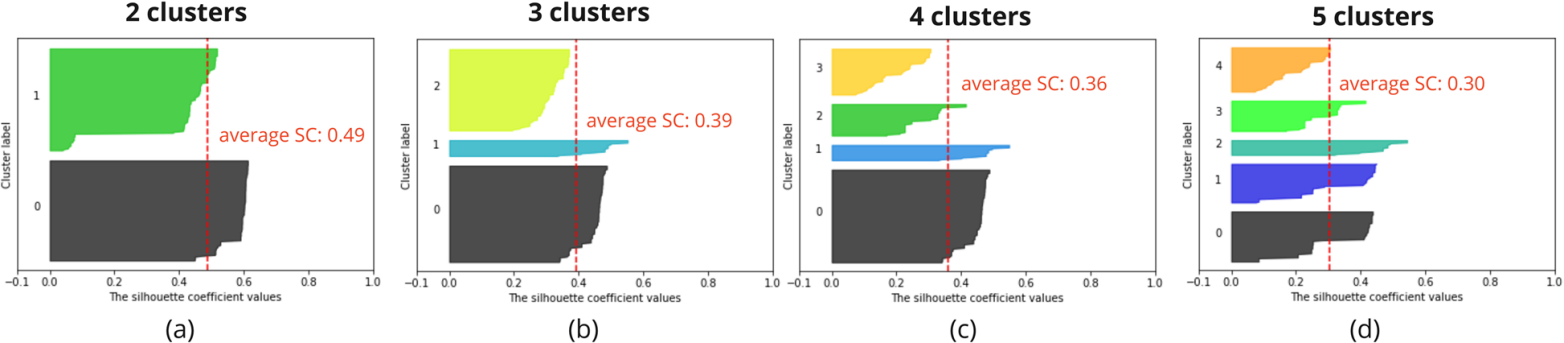
The hierarchical clustering of the action plans resulted in 3 clusters. The Silhouette Analysis for this clustering indicates that 3 clusters lead to the best clustering of the data (b). SC = silhouette coefficient

Figure 3 gives an overview of the Silhouette Analysis for the hierarchical clustering of the action plans for a range of 2 to 5 clusters. The Silhouette Analysis for this clustering indicates that 3 clusters lead to the best clustering of the data (b). Even though the average SC of 2 clusters is higher (a), cluster 1 contains some samples with a significantly lower SC. It can be assumed that when splitting in 3 clusters, cluster 1 of (a) is split into cluster 1 and cluster 2 of (b). For a higher number of clusters, the average SC decreases (c)(d).

**Fig. 4:**
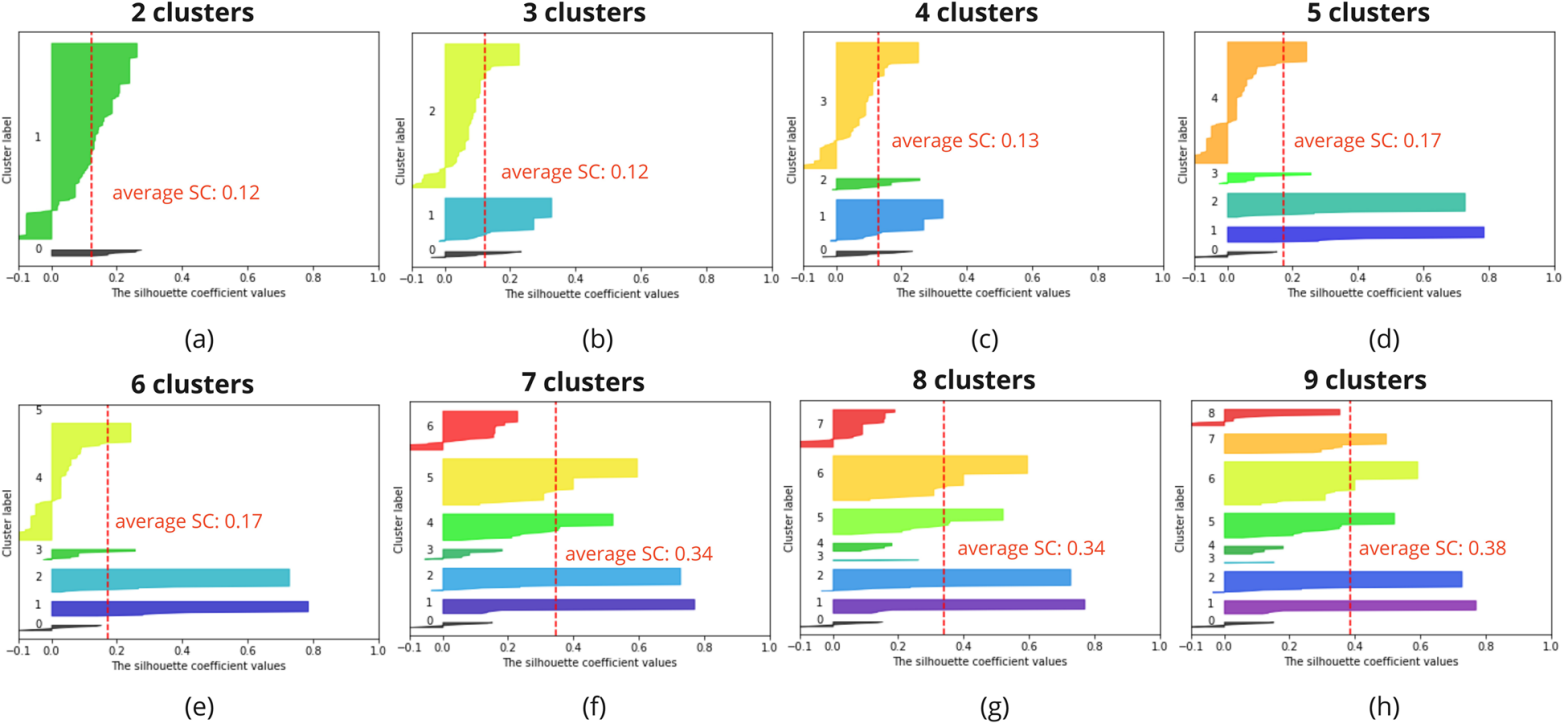
The hierarchical clustering of the coping plans resulted in 8 clusters. The Silhouette Analysis for this clustering indicates that 8 clusters lead to the best clustering of the data (g). SC = silhouette coefficient

Figure 4 shows the results of the Silhouette Analysis for the hierarchical clustering of the coping plans for 2 to 9 clusters. The clustering resulted in 8 clusters of coping plans. The Silhouette Analysis indicates a higher SC for a higher number of clusters (f-h) and a SC close to zero for 6 clusters or less (a-e). For 7 clusters, 4 clusters contain samples with a negative SC (f), indicating wrongly clustered samples, whereas for 8 or 9 clusters, this is reduced to 3 clusters containing samples with a negative SC (g,h). 8 clusters is the preferred cluster number (g), as the size of the clusters varies less compared to the size of the clusters in the case of 9 clusters. Moreover, the SC drops for the smaller clusters (h). However, it has to be noted that the data set used for clustering is limited in size and is not perfect, i.e. the dataset can contain outliers, for which more and smaller clusters will lead to a more optimal result. Nonetheless, fewer clusters, each containing more samples can result in more robust clusters to avoid tailoring to outliers.

**Fig. 5:**
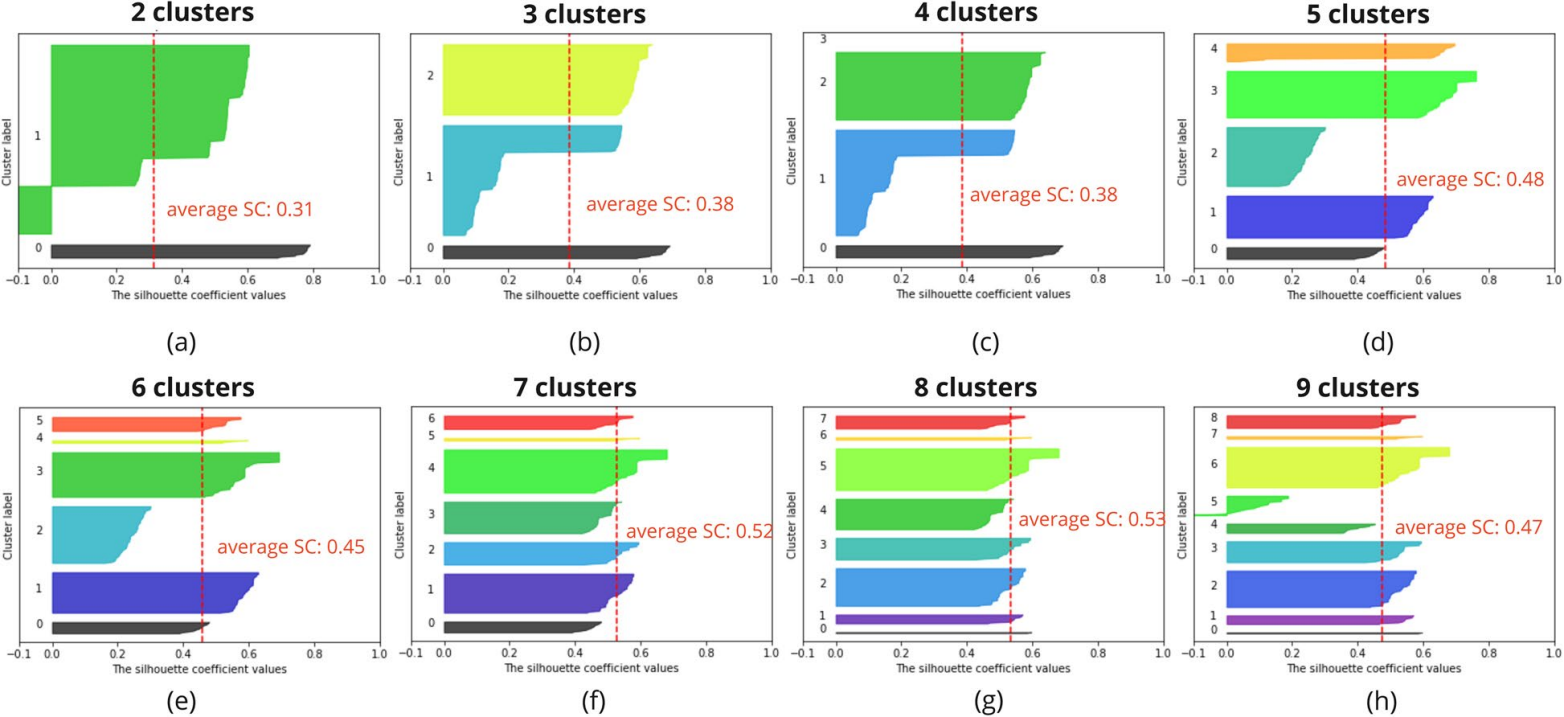
The hierarchical clustering of the combination of action and coping plans resulted in 8 clusters. The Silhouette Analysis for this clustering indicates that 8 clusters lead to the best clustering of the data (g). SC = silhouette coefficient

Similarly, Fig. 5 shows the results of the hierarchical clustering of the combination of action and coping plans for 2 to 9 clusters. The results indicate the highest average SC for 8 clusters, namely 0.53 (g). All clusters have a score equal or higher to the average SC and overall size of the clusters is similar, containing no samples with a negative SC. The hierarchical clustering for the combination of the action and coping plans resulted in 8 clusters, which is confirmed by the results of the Silhouette Analysis.

#### Statistical analysis

After identification of the clusters, they were imported in SPSS 26. Chi^2^-tests and analyses of variances were executed to examine the relations between the identified clusters with the specific user information (i.e. age, BMI, gender, educational level, occupation, marital status and motivational stage). For the continuous variables age and BMI, Tukey Post-Hoc tests were used. *P*-values of less than 0.05 were considered statistically significant.

## Results

### Clusters of action plans

**Fig. 6:**
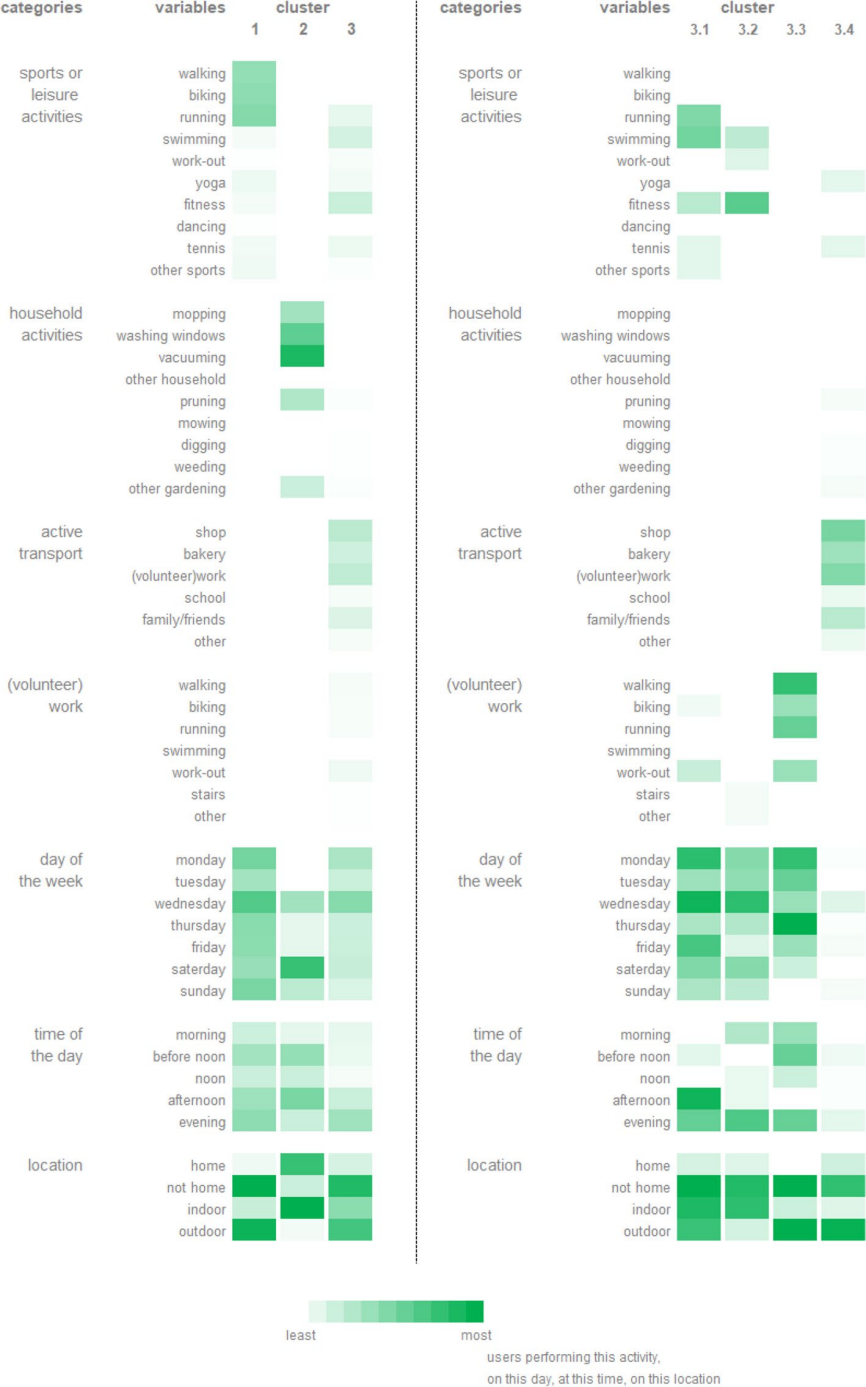
Clusters of action plans (left) and Subclusters of action plan cluster 3 (right). The first column represents the overarching categories of the variables of the action plan. The second column represents all variables of the action plan which were used to identify the clusters. The other columns represent the identified clusters which are each characterized by the different variables of the action plan. The greener the variable of the action plan, the more users performed this activity, did the activity on this day of the week, at this time of the day, or on this location

Three clusters of action plans were identified through the hierarchical clustering. Cluster 1 consisted of 110 action plans, created by 33 individuals. Cluster 2 consisted of 19 action plans, created by 8 individuals. Cluster 3 consisted of 93 action plans, created by 32 individuals. Each cluster could be characterized by variables of the action plan, as visualized in Fig. 6 (left side). Cluster 1 was characterized by the sports or leisure activities walking, biking and running. These could be performed on every day of the week and at any time of the day. The activities mainly took place outside and not at home. Cluster 2 was characterized by household activities (i.e. vacuuming, cleaning windows, mopping) which mainly took place on Saturdays. These activities evidently took place at home and inside. Cluster 3 was characterized by active transport and different sports or leisure activities (i.e. fitness, swimming, running, tennis) which could be performed on every day of the week and mainly took place in the evening. These activities did not take place at home and could be inside or outside.

#### Subclusters of action plans

To explore the clusters of action plans into more detail, additional analyses were performed and can be found in Additional file 2. As a result, subclusters of the three clusters of action plans were identified. Four subclusters of cluster 1, three subclusters of cluster 2 and four subclusters of cluster 3 of action plans were identified. The subclusters of cluster 1 and 2 were not considered to be relevant since they only divided the activities walking, biking and running (for cluster 1) and different household activities (for cluster 2). Therefore, only the subclusters of cluster 3 are presented in this paper (Fig. 6, right side). In short, cluster 3.1 was characterized by different sport activities, cluster 3.2 mainly by the activity ‘fitness’, cluster 3.3 by activities related to work and cluster 3.4 was characterized by active transport.

#### Linking (sub) clusters of action plans to user information

Some differences in user information between clusters of action plans were found. Significant differences were found in age, BMI, gender, occupation and motivational stage (Table 2). Pairwise comparison showed that individuals creating action plans of cluster 3 were significantly younger (Mean = 31.46; SD = 11.12) than individuals creating action plans of cluster 1 (Mean: 39.48; SD = 17.08) (*P* < .001) and cluster 2 (Mean = 44.11; SD = 14.09) (*P* = .002). Furthermore, individuals creating action plans of cluster 1 had a significantly higher BMI (Mean = 25.71; SD = 3.63) than individuals creating action plans of cluster 2 (Mean = 23.67; SD = 3.92) (*P* = .034) and 3 (Mean = 24.04; SD = 2.63) (*P* < .001). Individuals creating action plans of cluster 2 were likely to be women, to have a job and to be pre-intenders or intenders for PA. All pairwise comparisons can be found in Additional file 3.

**Table 2:** Differences in user information between clusters of action plans

		Cluster 1 (n_**p**_ = 33, n_**ap**_ = 110)	Cluster 2 (n_**p**_ = 8, n_**ap**_ = 19)	Cluster 3 (n_**p**_ = 32, n_**ap**_ = 93)	Significance of difference	
Clusters of action plans		Biking, walking, running | Every day | All times | Not home | Outside	Household activities | Saturday | At home | Inside	Active Transport & Different sports | Every day | Evening | Not home | Inside & outside	F/ X^**2**^	***P***-value
**Age**	Mean ± SD	39.48 ± 17.08 ^c^	44.11 ± 14.09 ^c^	31.46 ± 11.12 ^a,b^	**14.40**	**< 0.001**
**BMI**	Mean ± SD	25.71 ± 3.63 ^b, c^	23.67 ± 3.92 ^a^	24.04 ± 2.63 ^a^	**13.40**	**< 0.001**
**Gender**	% female	52%	100%	65%	**16.92**	**< 0.001**
**Education**	% higher education level	67%	89%	69%	3.87	0.145
**Occupation**	% having a job	40%	79%	40%	**10.79**	**0.004**
**Marital status**	% having no partner	46%	53%	58%	2.77	0.250
**Stage**	% (pre)intender	45%	100%	48%	**20.34**	**< 0.001**

Results of analysis of variance and chi-square tests. Superscript letters and bold p-values represent significant differences between clusters. ^a^ significantly different from cluster 1, ^b^ significantly different from cluster 2; ^c^ significantly different from cluster 3. n_p_ = number of individuals, n_ap_ = number of action plans

BMI was significantly different between the subclusters of cluster 3 (Table 3). Pairwise comparison showed that individuals creating action plans of cluster 3.4 had a significantly higher BMI (Mean = 25.07; SD = 2.26) than individuals creating action plans of cluster 3.1 (Mean = 22.40; SD = 1.38) (*P* < .001) and 3.3 (Mean = 21.08; SD = 1.65) (*P* = .003).

**Table 3:** Differences in user information between subclusters of action plan cluster 3

Subclusters of action plan cluster 3		Cluster 3.1 (n_**p**_ = 8, n_**ap**_ = 18)	Cluster 3.2 (n_**p**_ = 9 n_**ap**_ = 23)	Cluster 3.3 (n_**p**_ = 4, n_**ap**_ = 5)	Cluster 3.4 (n_**p**_ = 2, n_**ap**_ = 20)	Significance of difference	
		Different sports | Monday, Wednesday, Friday | Afternoon, evening | Not home | Inside, Outside	Fitness & swimming | Wednesday | Evening | Not home | Inside	Work | During week | Morning, (before) noon | Not home | Outside	Active Transport | Not home | Outside	F/X^**2**^	***P***-value
**Age**	Mean ± SD	30.67 ± 7.64	28.35 ± 10.25	30.80 ± 7.16	33. 36 ± 12.73	2.10	0.15
**BMI**	Mean ± SD	22.40 ± 1.38 ^d^	23.86 ± 3.11	21.08 ± 1.65 ^d^	25.07 ± 2.26 ^a,c^	**14.74**	**< 0.001**
**Gender**	% female	61%	78%	80%	60%	3.01	0.390
**Education**	% higher education level	56%	61%	80%	77%	3.77	0.288
**Occupation**	% having a job	56%	35%	40%	36%	2.37	0.500
**Marital status**	% having no partner	50%	78%	40%	53%	5.46	0.141
**Stage**	% (pre)intender	56%	39%	60%	49%	1.44	0.697

Results of analysis of variance and chi-square tests. Superscript letters and bold *p*-values represent significant differences between clusters. ^a^ significantly different from cluster 3.1, ^b^ significantly different from cluster 3.2, ^c^ significantly different from cluster 3.3, ^d^ significantly different from cluster 3.4. n_p_ = number of individuals, n_ap_ = number of action plans

### Clusters of coping plans

**Fig. 7:**
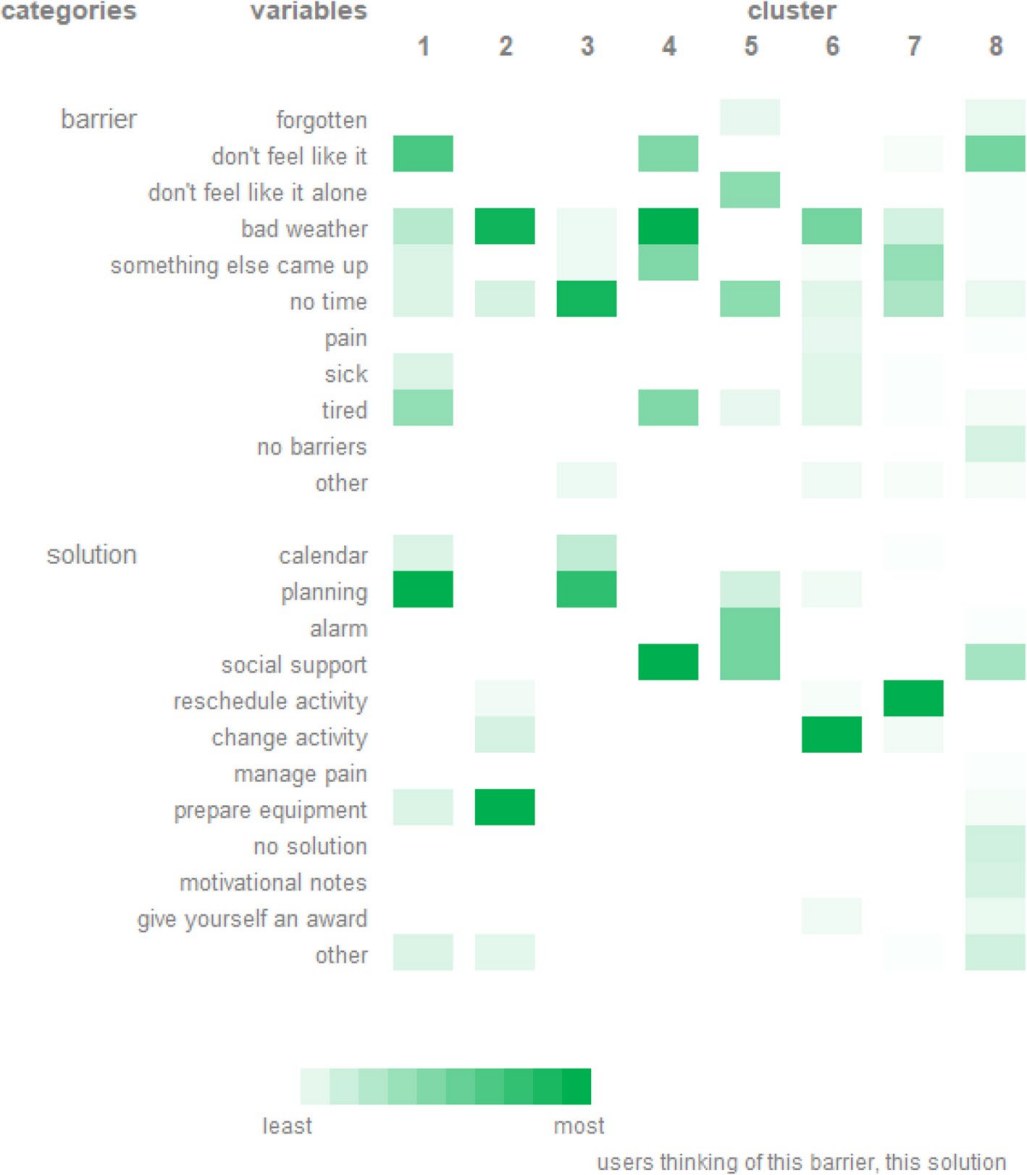
Clusters of coping plans. The first column represents the overarching categories of the variables of the coping plan. The second column represents all variables of the coping plan which were used to identify the clusters. The other columns represent the identified clusters which are each characterized by the different variables of the coping plan. The greener the variable of the coping plan, the more users thought about this barrier and this solution

Eight clusters of coping plans were identified. Each cluster was characterized by a barrier of the coping plan and a solution of the coping plan, which means that relatively consistent pairs of barriers with their solutions could be identified (Fig. 7). For example, cluster 2 consisted of 18 coping plans, made by 15 individuals and was characterized by the barrier “bad weather” and the solution “prepare equipment”. Furthermore, it is important to note that the majority of these clusters were characterized by other pairs of barriers and solutions than proposed by the questions and answer options in the intervention of MyPlan 2.0 (see Fig. 2).

#### Linking clusters of coping plans to user information

Some differences in user information between clusters of coping plans were found. Significant differences were found in education and motivational stage (Additional file 3). For example, individuals creating coping plans of cluster 4 *(*i.e. *barrier = bad weather; solution = social support*), 7 *(*i.e. *barriers = something else came up & no time; solution = reschedule activity)* and 8 *(*i.e. *barrier = don’t feel like it; solution = social support)* were more likely to have a lower educational level. Individuals creating coping plans of clusters 4 (i.e. *barrier = bad weather; solution = social support*) were pre(intenders). However, linking clusters of coping plans in general to user information did not appear to be straightforward as most of the significant findings lack a logical explanation (e.g. the link between having a lower educational level and creating a coping plan containing the barrier ‘bad weather’ and solution ‘social support’ might not be logically explained).

### Clusters of the combination of action and coping plans

**Fig. 8:**
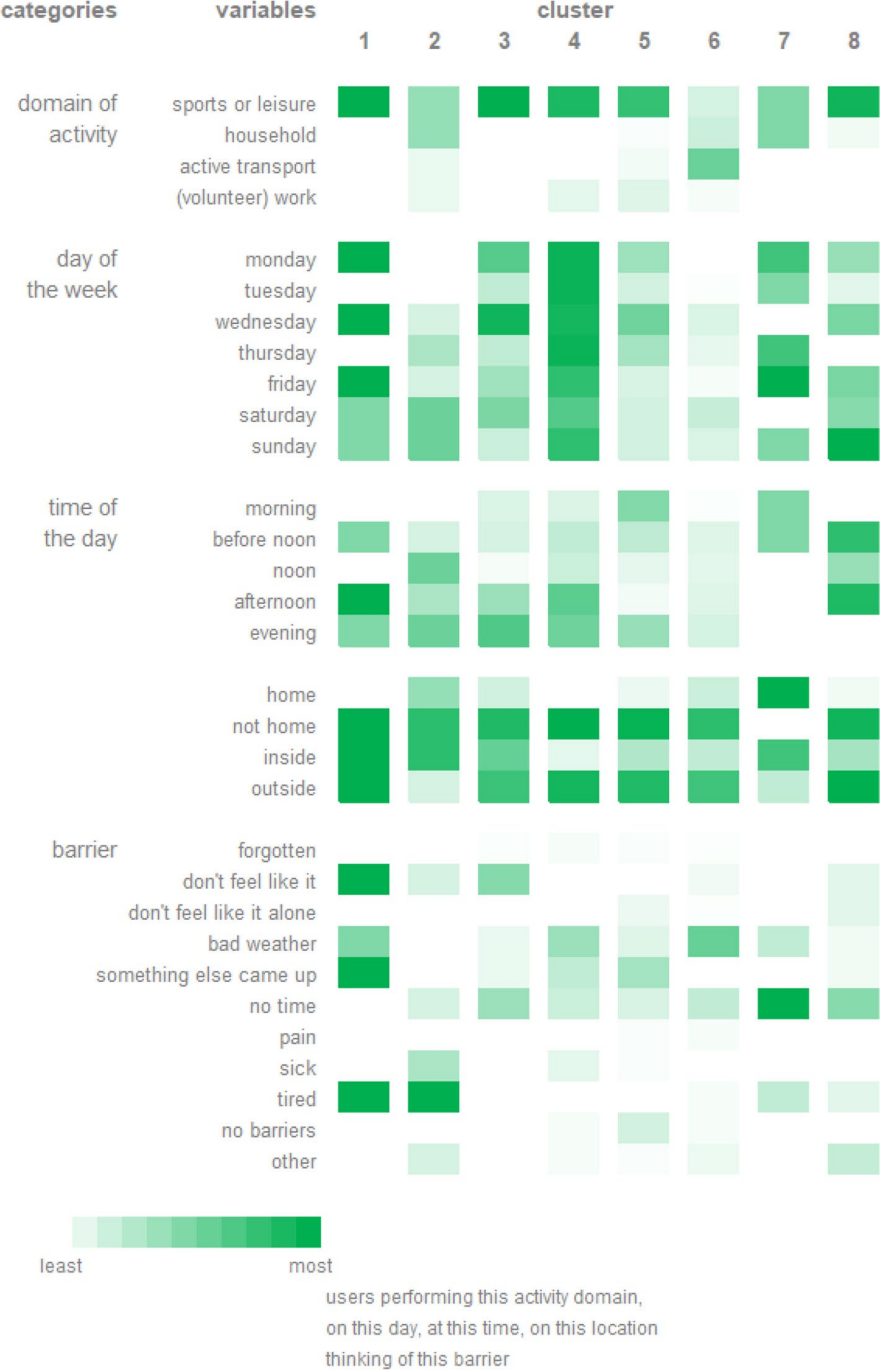
Clusters of combinations of action and coping plans. The first column represents the overarching categories of the variables of the action and coping plan. The second column represents all variables of the action plan and the barriers of the coping plan which were used to identify the clusters. The other columns represent the identified clusters which are each characterized by the different variables of the action plan and the barriers of the coping plan. The greener the variable of the action and coping plan, the more users performed this activity domain, on this day, at this time, on this location, and thought about this barrier

Eight clusters of the combination of action and coping plans were identified. Each cluster was characterized by different variables of the action plan and by one or two barriers of the coping plan (see Fig. 8). For example, cluster 6 consisted of 53 combinations of action and coping plans, created by 23 individuals. The cluster was characterized by “active transport” and the barrier “bad weather”.

#### Linking clusters of combination of action and coping plans to user information

Differences in user information between clusters of the combination of action and coping plans were found. Significant differences were found for gender, education, occupation, marital status and motivational stage (Additional file 3). For example, individuals creating action and coping plans of cluster 1 (i.e. *action plan = sport or leisure, weekdays, afternoon, not home; barrier = tired & don’t feel like it, something else came up*), 3 (i.e. *action plan = sports or leisure, on Monday and Wednesday, evening, not home; barrier = don’t feel like it, no time*) and 8 (i.e. *action plan = doing sports or leisure activities, on Sunday, before noon and afternoon, not home and outside, barrier = no time*) were less likely to have a partner. However, linking these clusters of combinations of action and coping plans in general to user information did not appear to be straightforward as all these significant findings lack a logical explanation.

## Discussion

The study explored the feasibility of applying a clustering method to develop a knowledge-base, which might be a first step towards more personalized suggestions on the content level in future digital health interventions. More specifically, this study investigated whether user information was related to specific action and coping plans. The results can be readily summarized. First, we were able to cluster action plans, coping plans and the combination of action and coping plans. Second, relating these clusters to user information was possible for action plans, but proved more difficult for the coping plans and specific combination of action and coping plans.

Our study revealed that some user characteristics related toward specific action plans. 1) Users with a higher BMI were more likely to choose outdoor leisure activities (walking, biking, running). 2) Women, users that did not perform PA regularly yet, or users who had a job, were more likely to choose for household activities. 3) Younger users were more likely to choose for active transport and different sports activities (fitness, swimming, tennis). Of these younger adults, users with a higher BMI were more likely to choose for active transport whereas users with a lower BMI would choose for different sports or work-related activities. Overall, these findings suggest that with the approach used in this study, it is feasible to find relations between action plans and specific personal user information. Consequently, the knowledge acquired from these findings might be used to define relationships in a knowledge-base and to ultimately personalize suggestions for action plans.

Although we could identify relatively consistent pairs of barriers and solutions formulated in the clusters of coping plans, we concluded that no logical link was found between user information and coping plans or specific combinations of action and coping plan. This was concluded since these results could not be compared to previous studies, nor theory. The reason that no logical link was found might be due to the fact that 8 clusters were identified for both coping plans and for specific combinations of action and coping plans. This is a relatively large number of clusters to relate user information to and may explain why, although statistical differences were found, these differences were not straightforward to interpret. Moreover, clusters of the specific combinations of action plans and coping plans were not considered to be valuable because it was difficult to meaningfully distinguish one cluster from another. Advanced clustering techniques with a larger and more heterogeneous sample may identify more valuable clusters in future research [30]. Also, the reason for the large numbers of clusters might reflect the fact that it is not possible to cluster coping plans or specific combinations of action and coping plans. In that case, only suggestions of action plans could be formulated rather than suggestions of coping plans or combinations of both. Another, maybe more important explanation, might be that the current paper only analyzed personal user information (i.e. demographic information and motivational stage). Consequently, relationships of other user information with plan characteristics remain unexplored. As it is known that PA is not a stable but dynamic (i.e. time-dependent) behaviour that varies throughout the day and from day to day [31, 32], it is more likely that barriers to certain physical activities, that are in a sense more hypothetical than action plans, relate more to contextual (e.g. the weather) and dynamic user information (e.g. emotions) than personal and rather stable user information (e.g. demographic information). Thus, future research should investigate whether more contextual and dynamic user information relates more strongly towards plan characteristics.

Considering the above findings, the clustering method used in the current study might be a feasible approach to acquire knowledge for a knowledge-base. However more user information will be needed to deliver personalized suggestions on the content level. First, we will illustrate how the results of the current study might be used to develop a knowledge-base, and then we will discuss what other user information might be needed to deliver more context-aware personalized suggestions. To develop a knowledge-base, acquired knowledge should be organized into a structure. Ontologies are one of the most popular approaches to structure these knowledge-bases as they are well specified [33], and can be combined with intelligent algorithms, which makes it possible to deliver personalized suggestions (e.g. for example, a user who does not perform PA regularly yet may get a suggestion to do a household activity). Furthermore, Larsen [33] shows that ontologies are already increasingly used by behavioural scientists. Indeed, several ontologies in the PA and behaviour change domain already exist [33]. For example, the Physical Activity Concept Ontology (PACO) structures different physical activities [34], the HAPA ontology structures all constructs of the HAPA-model [35], the Behaviour Change Interventions Ontology (BCIO) is a broader ontology that structures knowledge about interventions, their contexts, effects and evaluations [36, 37]. Even though ontologies such as the BCIO and HAPA ontology provide structures and their relationships on an abstract construct level (e.g. ‘intention’ influences ‘planning’, ‘planning’ positively influences the ‘Intenders’ to ‘Actors’ transitions relationship [35]), they still lack detailed concretization of these constructs and their interrelations at the content level [33]. Yet, ontologies with detailed concretizations at the content level are needed to deliver context-aware personalized suggestions. The current paper provides an approach to acquire knowledge for such an ontology. Here follows an example of how an ontology with the findings from the current paper may be used to deliver personalized suggestions. Suppose Rosy (older woman, higher BMI, high education, pre-intender for PA) logs into an m-health intervention. Which suggestions could the system deliver to get her more active the following week? Since the ontology contains relationships between user information and plan characteristics, the system can exploit the ontology with the help of intelligent algorithms and deduce which specific plan suggestions would match Rosy’s user profile. Based on the findings of the current paper, the suggestions ‘do a household activity’ or ‘do an outdoor activity such as walking, biking or running’ could be delivered to Rosy. In addition, Larsen [33] highlights the importance of combining ontologies with other ontologies in the field and asks the scientific community to update ontologies as new evidence emerges. As such, findings from the current paper could take other ontologies (such as the BCIO or the HAPA ontology) to a higher level by adding detailed concretizations at the content level. Consequently, the approach used in this study may contribute to the refinement of ontologies related to behaviour change interventions.

As previously stated, more user information in relation to plan characteristics should be acquired in order to develop a knowledge-base for context-aware personalized suggestions. Future studies might use the same approach of the current study to acquire more data from possible end users to shape the knowledge-base. However, the current approach should be enriched with other user information that might be placed on two continuums: First, relatively *stable user information* (i.e. information that does not change over a certain period of time) versus more *dynamic user information* (i.e. information that varies over a certain period of time). Second, *personal user information* (i.e. information related to the individual itself) versus *contextual user information* (i.e. information related to the context of the individual). The current paper addressed demographic info and motivational stage as *stable and personal user information*. Other personal and relatively stable user information worth investigating might be (perceived) motor skill competence or physical health. For example, recent research of Drenowatz [38] showed that a higher motor skill competence could be linked to more club sports participation. However these findings were only identified in children [38]. Another example shows that patients with chronic back pain perform more physical activities in the morning than in the evening compared to controls [39]. Second, exploring whether relatively *stable and contextual user information* relates to plan characteristics might provide important information as well. It might be interesting to explore whether users from various home or work environments make different plans or encounter different barriers to do PA. For example, research already showed that neighborhoods supporting a safe, enjoyable and social experience are associated with more leisure time walking among adults [40]. Third, as PA is not a static but dynamic behaviour [31, 32], it would be useful to examine whether certain *dynamic personal user information* (e.g. emotions, fatigue, pain) and *dynamic contextual user information* (e.g. weather, agenda of the day) relates to certain plan characteristics. This would enable the knowledge-base to deliver personalized suggestions based not only on relatively ‘stable information’ but also on ‘dynamic information’. For example, when the user has a busy day at work (*dynamic contextual info*), he might need other suggestions than when a user has more time; or when a user is stressed (*dynamic personal info*), the user might need other suggestions than when a user is more relaxed that day.

Some considerations should be taken into account for future research. To acquire more dynamic information, future studies should collect data on a smaller timeframe (day to day or even within days) as compared to the current study (only once at the start of the study). Relatively stable information might still be collected at the start of the study and/or at another moment depending on the study length (e.g. motivational stage may change after 3 weeks in an intervention). To acquire more dynamic information, ecological momentary assessment (EMA) might be used because this method makes it possible to collect real-time data based on repeated measures and observations that take place in participants’ daily environment [41]. For instance, during a 7-day EMA study the emotional state of a participant might be asked for example every 3 to 4 h, together with the question to make a PA plan for these following hours. This example would make it possible to relate certain emotions toward specific plans.

If a knowledge-base were to be developed based on the approach of the current study and the above-mentioned suggestions, future interventions to promote PA can exploit the knowledge-base in order to deliver context-aware personalized suggestions. Notwithstanding, these future interventions might take further steps toward context-aware personalized suggestions. First, despite the fact that more information in the knowledge-base may result in more context-aware personalized suggestions, one should be careful with asking too many questions at the start of a personalized intervention (in order to determine the new user’s profile). Using smart technologies such as wearables and apps (e.g. to measure stress, PA level, location, agenda, weather) could limit the number of questions. Second, the current study highlights the importance of a knowledge-base to deliver context-aware personalized suggestions in e-and m-health interventions. Nonetheless, it is unlikely that this approach will fully capture the complexity of behaviour change to provide context-aware personalized suggestions. Other approaches could complement the approach used in the current study. One of these approaches is ‘reinforcement learning’ [42]. In its most basic level, the system learns by measuring a success criterion for a given suggestion: if the success criterion is met, the probability of suggesting this suggestion a second time increases [42]. For example, the success criterion can be based on the user’s rating for a certain suggestion, or the user’s behaviour after that suggestion (e.g. if a user gets a suggestion of a plan to go for a walk and the user eventually goes for a walk). More advanced derivations of reinforcement learning should be explored in future interventions, for example success criterions of similar users [42, 43]. Third, another approach that might complement the current approach is the systems ID approach. The approach used in this study is still a *‘nomothetic’ approach* (i.e. making ‘aggregated’ conclusions of relationships of user info and plan characteristics), whereas ‘*ideographic approaches’* might deliver more context-aware personalized suggestions (i.e. making individualized conclusions of relationships between user info and plan characteristics by examining within-person variation over time). The systems ID approach is an ‘ideographic approach’ and learns from run-in periods to provide personalized suggestions (e.g. for a certain user it might be better to suggest a walking activity on a weekend day, whereas for another user it might be better to suggest a walking activity on a weekday) [31, 42]. The disadvantage of such a run-in period is that no context-aware personalized suggestions can be delivered at the beginning of such an intervention (which is also the case when using reinforcement learning on its own).

### Strengths and limitations

This study has several strengths. First, the current study demonstrated a proof-of-concept (clustering method) which provides insights in how a DSs with a knowledge-base could be developed in order to deliver more context-aware personalized suggestions in future digital interventions. Until now, many studies use a black-box approach in which details about how support is generated in the DSs are unknown [44]. Furthermore, the few available studies that did employ DSs lack information on the use of behaviour change theories [43]. Second, acquired knowledge in knowledge-bases in previous studies is mostly expert driven [23, 24], whereas the current study was theory-driven and data-driven. This approach gave us the opportunity to get more insights in comparison with only expert knowledge. Nonetheless, we urge caution when using clustering algorithms on their own (e.g. giving suggestions of household activities only to women, may reinforce standard, normative and/or stereotypical patterns of behaviour). Therefore, expert consultation remains important.

This study also has a number of limitations. First, the user sample for the current study was small, clustering with action plans and coping plans of a larger and more heterogeneous sample will possibly give better insights. Second, this study focused on which *plans users created* in order to do PA, we did not measure the actual performance of the plan. Investigating user information in relation to performing actual PA may also provide useful insights for personalized suggestions (e.g. if a user is feeling stressed, what kind of physical activities does the user perform?). Future studies may also use EMA to collect this data. Third, the current study demonstrated proof-of-concept to acquire knowledge for a knowledge-base in order to provide more personalized suggestions on the content level, however it is not clear whether this approach will be more effective to promote PA than simpler tailoring approaches (e.g. tailoring on construct level, tailoring based on preferences of the individual). Future research might investigate which approaches are most effective to promote PA. Fourth, the focus here was whether user information related toward specific action and coping plans, in order to deliver personalized suggestions of these plans on the content level. Future studies might also consider other BCTs, such as self-monitoring (e.g. older users maybe relate to other self-monitoring methods than younger users) or outcome-expectancies (e.g. when someone is stressed that person might need another message to see the advantage of PA than when someone is relaxed). Finally, the content and clusters of action and coping plans were based on data obtained from a digital health intervention. We do not expect that data from analogue approaches would lead to different results, but this assumption requires further corroboration.

## Conclusions

Until now, attrition rates in digital health interventions to promote PA are high which might be due to the lack of context-aware personalized suggestions in these interventions. The approach used in the current study might be a feasible approach to acquire knowledge for a knowledge-base, however more data from possible end users should be acquired in future research. This might result in a first type of context-aware personalized suggestions on the content level, as the system can provide initial suggestions based on the knowledge-base when a PA intervention has just started and the system did not have time to learn about the user. Over time, suggestions could be refined based on other approaches like reinforcement learning. Moreover, this approach extended prior efforts to personalize digital health interventions by providing context-aware personalized suggestions on the content level rather than on the construct level.

## Supplementary Information

**Additional file 1.** Coding of action and coping plans into different variables.

**Additional file 2.** Additional Silhouette Analysis to identify subclusters of the three clusters of action plans.

**Additional file 3.** Differences in user information between clusters of action plans, clusters of coping plans, and the combination of action and coping plans.

## Data Availability

Data supporting the results reported in this article are stored at the University of Ghent, Belgium. The datasets used during the current study are available from the corresponding author on reasonable request.

## Abbreviations

BCIO: behaviour change interventions ontology
BCT: behaviour change technique
DSs: decision support system
EMA: ecological momentary assessment
HAPA: health action process approach
PA: physical activity
PACO: physical activity concept ontology
SC: silhouette coefficient

## References

[1] Warburton DER, Nicol CW, Bredin SSD (2006). Health benefits of physical activity: the evidence. Can Med Assoc J.

[2] Haskell WL, Blair SN, Hill JO (2009). Physical activity: health outcomes and importance for public health policy. Prev Med.

[3] WHO. Classification of digital health interventions v1.0. 2018. http://apps.who.int/iris/bitstream/handle/10665/260480/WHO-RHR-18.06-eng.pdf;jsessionid. Accessed 23 Sept 2021.

[4] Broekhuizen K, Simmons D, Devlieger R, van Assche A, Jans G, Galjaard S (2018). Cost-effectiveness of healthy eating and/or physical activity promotion in pregnant women at increased risk of gestational diabetes mellitus: economic evaluation alongside the DALI study, a European multicenter randomized controlled trial. Int J Behav Nutr Phys Act.

[5] Glasgow RE, McKay HG, Piette JD, Reynolds KD (2001). The RE-AIM framework for evaluating interventions: what can it tell us about approaches to chronic illness management?. Patient Educ Couns.

[6] Vandelanotte C, Müller AM, Short CE, Hingle M, Nathan N, Williams SL, et al. Past, present, and future of eHealth and mHealth research to improve physical activity and dietary behaviors. J Nutr Educ Behav. 2016;48(3):219–228.e1. 10.1016/j.jneb.2015.12.006 26965100

[7] Webb TL, Joseph J, Yardley L, Michie S. Using the internet to promote health behavior change: a systematic review and Meta-analysis of the impact of theoretical basis, use of behavior change techniques, and mode of delivery on efficacy. J Med Internet Res. 2010;12(1):e4. 10.2196/jmir.1376 PMC2836773 20164043

[8] Schwarzer R, Lippke S, Luszczynska A (2011). Mechanisms of health behavior change in persons with chronic illness or disability: the health action process approach (HAPA). Rehabil Psychol.

[9] Karoly P (1993). Mechanisms of self-regulation: a systems view. Annu Rev Psychol.

[10] Schwarzer R, Luszczynska A (2008). How to overcome health-compromising behaviors - the health action process approach. Eur Psychol.

[11] Sniehotta FF, Scholz U, Schwarzer R (2005). Bridging the intention-behaviour gap: planning, self-efficacy, and action control in the adoption and maintenance of physical exercise. Psychol Health.

[12] Sutton S (2008). How does the health action process approach (HAPA) bridge the intention-behavior gap? An examination of the model’s causal structure. Appl Psychol.

[13] Lippke S, Ziegelmann JP, Schwarzer R (2005). Stage-specific adoption and maintenance of physical activity: testing a three-stage model. Psychol Sport Exerc.

[14] Schroé H, Van Dyck D, De Paepe A, Poppe L, Loh WW, Verloigne M (2020). Which behaviour change techniques are effective to promote physical activity and reduce sedentary behaviour in adults: a factorial randomized trial of an e- and m-health intervention. Int J Behav Nutr Phys Act.

[15] Poppe L, De Bourdeaudhuij I, Verloigne M, Shadid S, Van Cauwenberg J, Compernolle S (2019). Efficacy of a self-regulation-based electronic and Mobile health intervention targeting an active lifestyle in adults having type 2 diabetes and in adults aged 50 years or older: two randomized controlled trials. J Med Internet Res.

[16] Degroote L, De Paepe A, De Bourdeaudhuij I, Van Dyck D, Crombez G (2021). Effectiveness of the mHealth intervention ‘MyDayPlan’ to increase physical activity: an aggregated single case approach. Int J Behav Nutr Phys Act.

[17] Van der Mispel C, Poppe L, Crombez G, Verloigne M, De Bourdeaudhuij I (2017). A self-regulation-based eHealth intervention to promote a healthy lifestyle: investigating user and website characteristics related to attrition. J Med Internet Res.

[18] Schroé H, Crombez G, De Bourdeaudhuij I, Van Dyck D. Investigating When, Which, and Why Users Stop Using a Digital Health Intervention to Promote an Active Lifestyle: Secondary Analysis With A Focus on Health Action Process Approach–Based Psychological Determinants. JMIR Mhealth Uhealth. 2022;10(1):e30583. 10.2196/30583 PMC8845016 35099400

[19] Hekler E, Tiro JA, Hunter CM, Nebeker C (2020). Precision health: the role of the social and behavioral sciences in advancing the vision. Ann Behav Med.

[20] Schwarzer R, Cao DS, Lippke S (2010). Stage-matched minimal interventions to enhance physical activity in Chinese adolescents. J Adolesc Health.

[21] Poppe L, Van der Mispel C, Crombez G, De Bourdeaudhuij I, Schroe H, Verloigne M. How users experience and use an eHealth intervention based on self-regulation: mixed-methods study. J Med Internet Res. 2018;20(10):e10412. 10.2196/10412 PMC6231831 30274961

[22] Degroote L, Van Dyck D, De Bourdeaudhuij I, De Paepe A, Crombez G (2020). Acceptability and feasibility of the mHealth intervention ‘MyDayPlan’ to increase physical activity in a general adult population. BMC Public Health.

[23] Çelik Ertuğrul D, Elçi A. A survey on semanticized and personalized health recommender systems. Expert Systems. n/a(n/a):e12519.

[24] Pratiwi PS, Xu Y, Li Y, Trost SG, Clanchy K, Tjondronegoro DW. User profile ontology to support personalization for e-coaching systems. In: International workshop on ACM international conference on information and knowledge management (CIKM). Turin; 2018.

[25] Schroe H, Van der Mispel C, De Bourdeaudhuij I, Verloigne M, Poppe L, Crombez G. A factorial randomised controlled trial to identify efficacious self-regulation techniques in an e- and m-health intervention to target an active lifestyle: study protocol. Trials. 2019;20(1):340. 10.1186/s13063-019-3456-7 PMC6558816 31182147

[26] Thomas S, Reading J, Shephard RJ (1992). Revision of the physical activity readiness questionnaire (PAR-Q). Can J Sport Sci.

[27] Pedregosa F, Varoquaux G, Gramfort A, Michel V, Thirion B, Grisel O (2011). Scikit-learn: machine learning in {P}ython. J Mach Learn Res.

[28] Waggener W (1995). Pulse Code Modulation Techniques.

[29] Rousseeuw PJ (1987). Silhouettes: a graphical aid to the interpretation and validation of cluster analysis. J Comput Appl Math.

[30] Saxena A, Prasad M, Gupta A, Bharill N, Patel OP, Tiwari A (2017). A review of clustering techniques and developments. Neurocomputing..

[31] Conroy DE, Hojjatinia S, Lagoa CM, Yang C-H, Lanza ST, Smyth JM (2019). Personalized models of physical activity responses to text message micro-interventions: a proof-of-concept application of control systems engineering methods. Psychol Sport Exerc.

[32] Martin KR, Koster A, Murphy RA, Van Domelen DR, Hung M-y, Brychta RJ (2014). Changes in daily activity patterns with age in U.S. men and women: National Health and nutrition examination survey 2003–04 and 2005–06. J Am Geriatr Soc.

[33] Larsen KR, Michie S, Hekler EB, Gibson B, Spruijt-Metz D, Ahern D (2017). Behavior change interventions: the potential of ontologies for advancing science and practice. J Behav Med.

[34] Kim H, Mentzer J, Taira R (2019). Developing a physical activity ontology to support the interoperability of physical activity data. J Med Internet Res.

[35] Hale J, Hastings J, West R, Lefevre CE, Direito A, Bohlen LC (2020). An ontology-based modelling system (OBMS) for representing behaviour change theories applied to 76 theories. Wellcome Open Res.

[36] Michie S, West R, Finnerty AN, Norris E, Wright AJ, Marques MM, et al. Representation of behaviour change interventions and their evaluation: development of the upper level of the behaviour change intervention ontology. Wellcome Open Res. 2021;5:123. 10.12688/wellcomeopenres.15902.1 PMC7868854 33614976

[37] Michie S, Thomas J, Johnston M, Mac Aonghusa P, Shawe-Taylor J, Kelly MP, et al. The human behaviour-change project: harnessing the power of artificial intelligence and machine learning for evidence synthesis and interpretation. Implement Sci. 2017;12(1):121. 10.1186/s13012-017-0641-5 PMC5648456 29047393

[38] Drenowatz C, Greier K (2019). Cross-sectional and longitudinal association of sports participation, media consumption and motor competence in youth. Scand J Med Sci Sports.

[39] van Weering MGH, Vollenbroek-Hutten MMR, Tönis TM, Hermens HJ (2009). Daily physical activities in chronic lower back pain patients assessed with accelerometry. Eur J Pain.

[40] Nehme EK, Oluyomi AO, Calise TV, Kohl HW (2016). Environmental correlates of recreational walking in the neighborhood. Am J Health Promot.

[41] Degroote L, DeSmet A, De Bourdeaudhuij I, Van Dyck D, Crombez G (2020). Content validity and methodological considerations in ecological momentary assessment studies on physical activity and sedentary behaviour: a systematic review. Int J Behav Nutr Phy.

[42] Phatak SS, Freigoun MT, Martín CA, Rivera DE, Korinek EV, Adams MA (2018). Modeling individual differences: a case study of the application of system identification for personalizing a physical activity intervention. J Biomed Inform.

[43] Cheung KL, Durusu D, Sui X, de Vries H (2019). How recommender systems could support and enhance computer-tailored digital health programs: a scoping review. Digital Health.

[44] Hors-Fraile S, Rivera-Romero O, Schneider F, Fernandez-Luque L, Luna-Perejon F, Civit-Balcells A (2018). Analyzing recommender systems for health promotion using a multidisciplinary taxonomy: a scoping review. Int J Med Inform.

