# Supplementary material for: Towards more personalized digital health interventions: a clustering method of action and coping plans to promote physical activity
Source: BMC Public Health. 2022 Dec 12;22:2325. doi: 10.1186/s12889-022-14455-4 (PMC9746174; doi:10.1186/s12889-022-14455-4)
Supplement: Supplementary file 2 — Additional file 2. Additional Silhouette Analysis to identify subclusters of the three clusters of action plans. [file 12889_2022_14455_MOESM2_ESM.pdf]

Additional file 2. Additional Silhouette Analysis to identify subclusters of the three clusters of action plans.

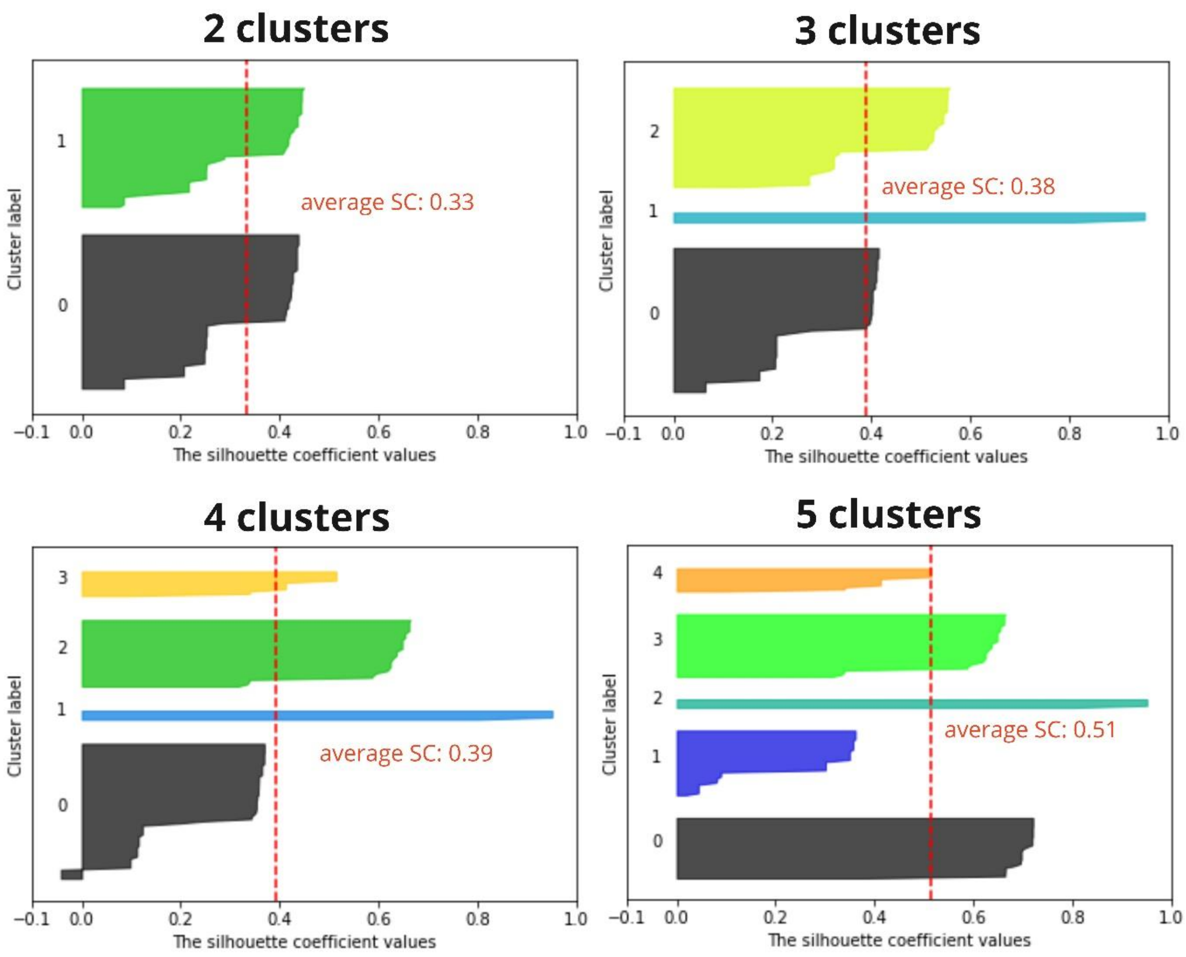

The hierarchical clustering of action plan cluster 1 resulted in 4 subclusters. The Silhouette Analysis for this clustering indicates that 4 clusters lead to the best clustering of the data. SC= silhouette coefficient.

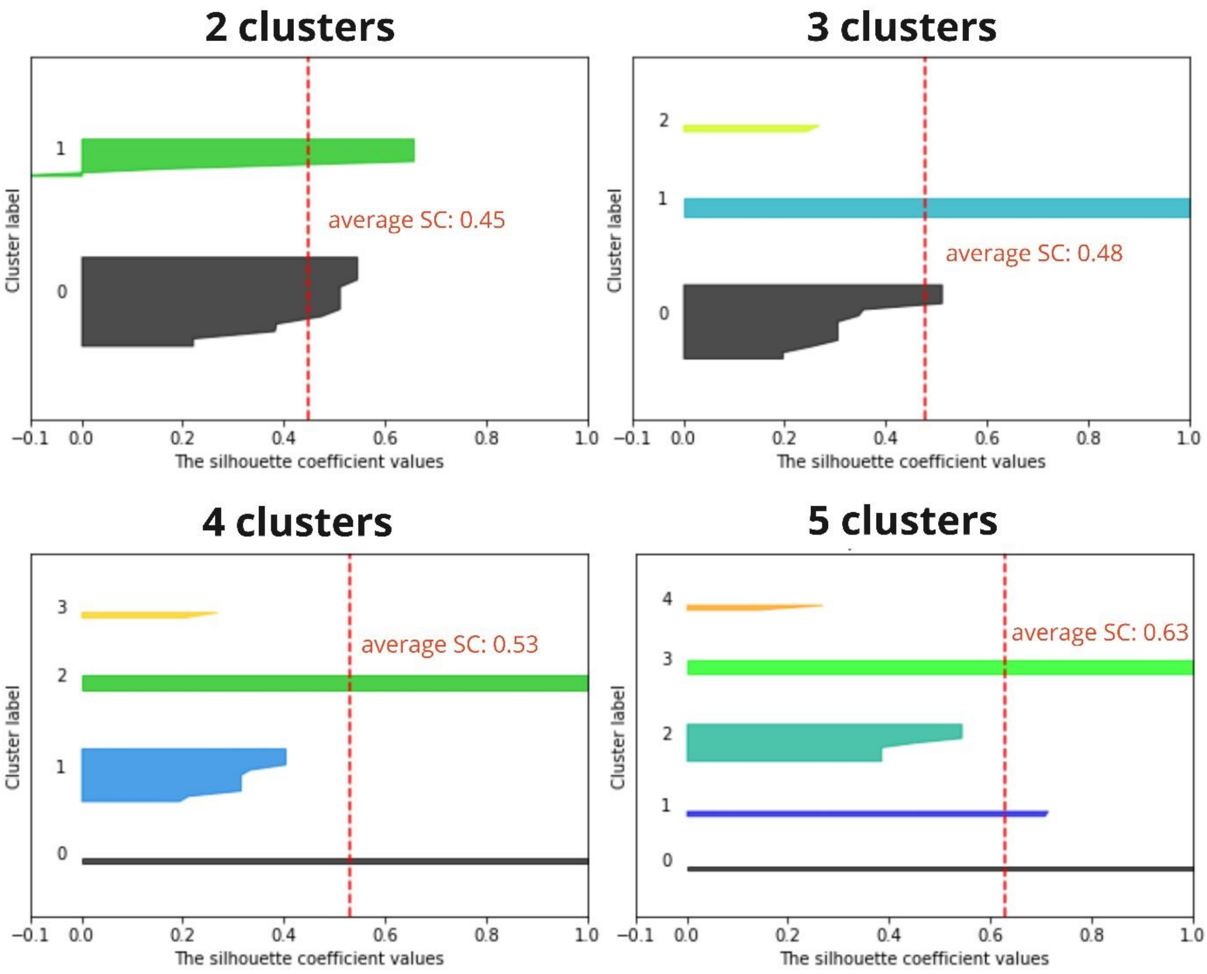

The hierarchical clustering of action plan cluster 2 resulted in 3 subclusters. The Silhouette Analysis for this clustering indicates that 3 clusters lead to the best clustering of the data. SC= silhouette coefficient.

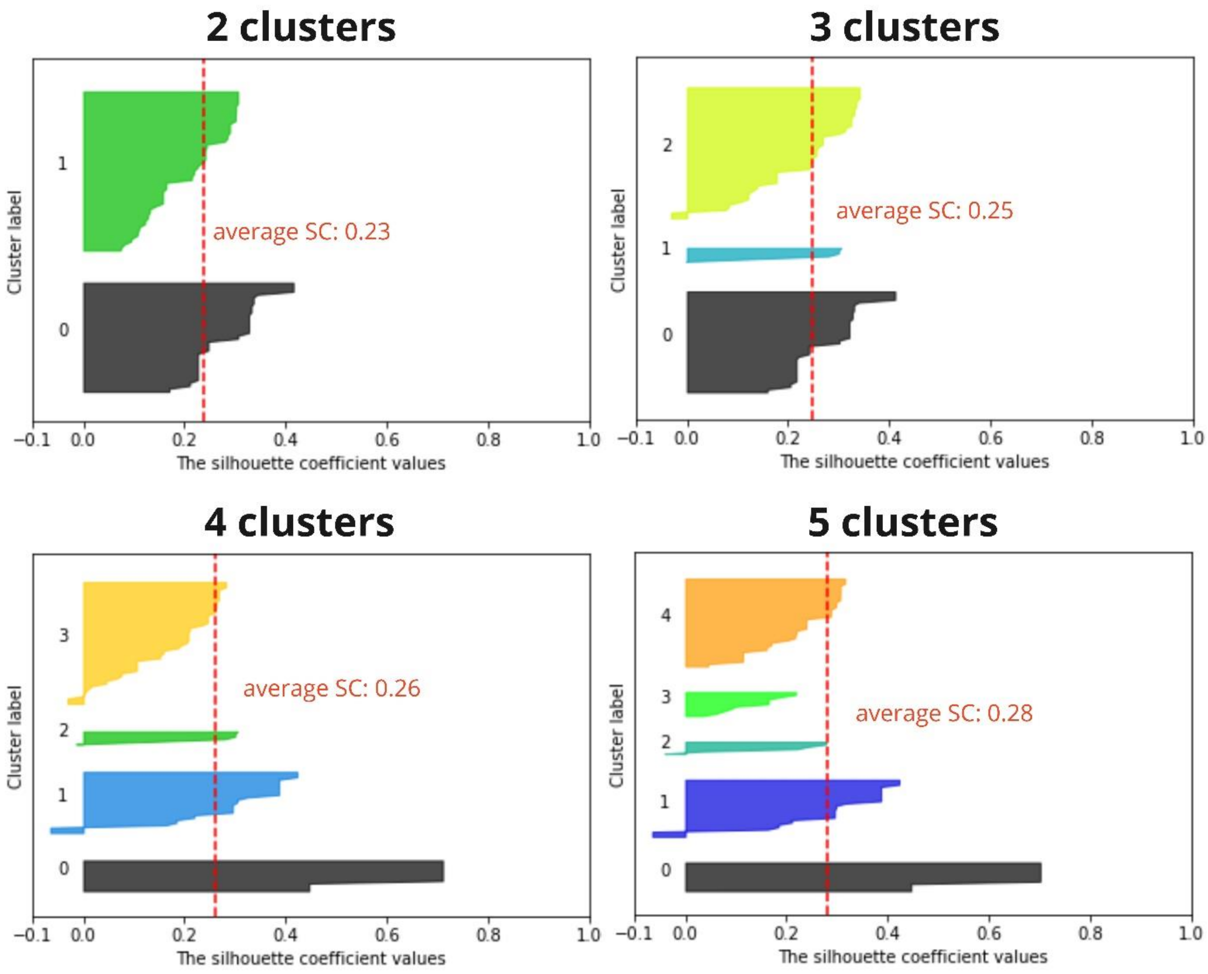

The hierarchical clustering of action plan cluster 3 resulted in 4 subclusters. The Silhouette Analysis for this clustering indicates that 4 clusters lead to the best clustering of the data. SC= silhouette coefficient.
